# Testing the Effects of 3D Multiple Object Tracking Training on Near, Mid and Far Transfer

**DOI:** 10.3389/fpsyg.2020.00196

**Published:** 2020-02-12

**Authors:** David J. Harris, Mark R. Wilson, Sarah J. R. Smith, Natalie Meder, Samuel J. Vine

**Affiliations:** ^1^School of Sport and Health Sciences, University of Exeter, Exeter, United Kingdom; ^2^Human Performance Team, Defence Science and Technology Laboratory Portsdown West, Fareham, United Kingdom

**Keywords:** NeuroTracker, working memory, cognitive training, sport, military

## Abstract

Cognitive training (CT) aims to develop domain general mental abilities to support functions like decision making, multitasking, and performance under pressure. Research to date has indicated that CT likely aids performance on lab-based cognitive tests, but there has been little demonstration of transfer to tasks representative of real-world high performance environments. This study aimed to assess transfer from a CT intervention to near and mid-level transfer tasks, plus a far transfer test representative of real-world multitasking in a military environment. 84 participants were randomized to four independent training groups, using *NeuroTracker*, a CT task based on 3D object tracking. There was no evidence for near transfer (to another object tracking task) or for far transfer to a route monitoring task designed to replicate real-world multitasking. There may, however, have been some improvement in working memory performance as a result of training. These findings raise further questions about whether domain general CT will transfer to real-world performance. Effective uses of CT may require more task specific training targeting mid-level transfer effects.

## Introduction

Cognitive training (CT) aims to develop domain-general mental abilities to improve performance in a range of daily activities. CT programmes consist of systematic practice on games, puzzles and tests designed to target cognitive functions such as working memory or attention (Simons et al., 2016). A major contention within the CT literature is whether any real evidence exists for true far transfer effects, that is, to tasks or situations beyond those employed during training (Owen et al., 2010; Stojanoski et al., 2018; Lintern and Boot, 2019). Nonetheless there has been considerable uptake of CT by sports teams, and a significant commercial market has developed around CT (estimated to be worth $8 billion by 2021; marketsandmarkets.com, 2017). CT has received particular interest from sporting and military researchers (Blacker et al., 2018; Walton et al., 2018), as these environments are considered to place significant demand on functions like working memory (WM) to support effective decision making (Furley and Memmert, 2010) and resisting performance breakdowns under pressure (Beilock and Carr, 2005; Ducrocq et al., 2018).

Cognitive training is based on the assumption that if cognitive abilities predict real-world performance and success, then practicing those abilities should improve performance in real-world tasks (Simons et al., 2016). Core cognitive abilities are indeed predictive of workplace performance (Ree et al., 1994; Schmidt and Hunter, 2004). Consequently, domain-general CT is particularly attractive as training payoffs could relate to multiple tasks and scenarios. Currently there is reliable evidence that CT, implemented in a variety of ways, leads to measurable improvements on cognitive tests that resemble the training method – that is, near transfer (Jaeggi et al., 2011; Morrison and Chein, 2011; Melby-Lervåg and Hulme, 2013; Sala and Gobet, 2019). It remains unclear, however, whether training transfers beyond the lab, to new tasks and contexts – that is, far transfer (Owen et al., 2010; Simons et al., 2016; Stojanoski et al., 2018; Sala and Gobet, 2019).

Many commercial methods of brain training, as well as bespoke applications used in research, are based on established cognitive tests, such as *n*-back working memory tests (e.g., Jaeggi et al., 2008, trained participants on an adaptive dual-*n*-back) or response-inhibition tests (e.g., see Biggs et al., 2015). Despite showing positive training effects on laboratory based cognitive tests, these approaches to CT have shown very little evidence of real-world transfer in healthy populations (Melby-Lervåg and Hulme, 2013; Harris et al., 2018; Stojanoski et al., 2018). Other methods featuring perceptual-cognitive elements have, however, produced more promising results. For instance, Ducrocq et al. (2016) and colleagues demonstrated the benefits of training inhibitory control using a visual search paradigm, leading to improvements in attention control and volleying performance in a pressurized tennis task (see also Biggs et al., 2015).

A CT task which has received particular interest from both sport and the military, and features a perceptual-cognitive component, is *NeuroTracker*, a 3-dimensional multiple object tracking (MOT) task. NeuroTracker training has been linked with improvements in working memory performance (Parsons et al., 2016; Vartanian et al., 2016) and attention (Tullo et al., 2018), but more importantly, has shown potential far transfer effects. Legault and Faubert (2012) found that NeuroTracker training improved biological motion perception, while Romeas et al. (2016) found improvement in blinded coach ratings of soccer passing performance and accuracy of decision-making. While these findings suggest transfer of training, the transfer tests used do not replicate the multitasking nature of many cognitively demanding sporting or military tasks. Consequently, we aimed to investigate whether NeuroTracker training would aid performance in a more representative task with objective performance outcomes.

In an extensive review of brain training research, Simons et al. (2016) identified particular methodological issues within the field, including; inadequate power justification and low sample size; combined training interventions to maximize the chance of an effect; a lack of transfer tests with real-world relevance; and an over reliance on self-report outcomes. We aimed to address these issues by testing a single training method (3D MOT), in an appropriately powered study, using a transfer task designed to represent real-world multitasking. Given the interest of practitioners in devices such as NeuroTracker, we also aimed to investigate practical issues relating to method of training delivery and number of training sessions. Consequently we compared the recommended NeuroTracker training delivery^1^ of 20 training “blocks” across five sessions, with an abbreviated training programme (three sessions), a portable method of training, and a passive control group. The portable and abbreviated training groups served as active controls for the full training delivery. Based on previous positive effects of adaptive object tracking training (Parsons et al., 2016; Romeas et al., 2016), it was predicted that NeuroTracker training would lead to improvements in a second object tracking task (near transfer), *n*-back working memory performance (mid-level transfer), and performance on a multitasking far transfer task.

## Materials and Methods

### Participants

Eighty four participants (50 female, mean age = 23.1 years, SD = 3.9) were recruited from an undergraduate population using poster advertising and word of mouth. Sample size determination was based on the effect size obtained by Romeas et al. (2016) (η_p_^2^ = 0.162), which indicated that, given α = 0.05, 20 participants per group were required to obtain power (1- β) of 0.90 in an independent groups design. Participants were randomly assigned to one of four training groups: *Full –* five sessions of NeuroTracker training on the full device; *Abbreviated –* three sessions on the full device; *Portable –* five sessions on a portable tablet version of the device; *Control –* no-training control group. The portable and abbreviated training groups served to investigate the importance of training duration and delivery method, and provided an active control for full NeuroTracker training. Ethical approval for the study was granted by the Ministry of Defence Research Ethics Committee and the University prior to the start of data collection. Participants gave written informed consent at the start of testing and were compensated £10.00 for participation.

### Materials

#### NeuroTracker Training Task

**FIGURE 1:**
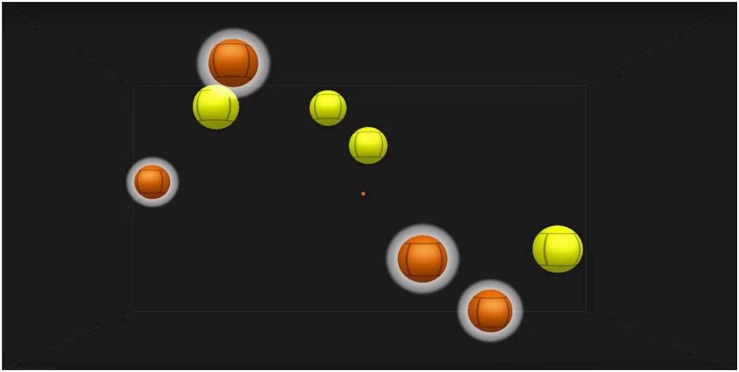
Screenshot of NeuroTracker task. Users are required to follow the temporarily highlighted targets.

NeuroTracker training consisted of four blocks of 20 object tracking trials per session. Each block lasts approximately 6 min. On each trial the user is presented with eight yellow balls (each ∼8 cm diameter, equivalent to 2° visual angle), four of which must be tracked (identified by flashing orange at the start of each trial), and four which should be ignored (Figure 1). The balls move in three dimensions around a virtual cube, bouncing off the sides. Each trial lasts 10 s (2 s identification phase, followed by 8 s of movement). The full version of the task was presented on a large screen (100 × 150 cm) using a 3D projector (Epson EHTW5650) and active 3D glasses (Epson ELPGS03). The portable version was presented on a 12.3 inch Microsoft Surface Pro tablet, using anaglyph 3D glasses. Trial speed was constantly adapted to provide an optimal level of challenge; if a correct response is given, speed increases and if an incorrect response is given, speed decreases (see Faubert and Sidebottom, 2012 for more detail). Performance on this task was assessed through the speed threshold metric provided by NeuroTracker software. The speed threshold score is the speed at which participants were able to identify all targets correctly 50% of the time. As trial speed is constantly adapted, this represents a more informative measure than number of correct items. Pre and post-performance assessment was based on two blocks of 20 tracking trials.

#### Near Transfer Task

Near transfer was assessed using a MOT task, based on that used by Jardine and Seiffert (2011). Stimuli were programmed in MATLAB (v2016a) using the Psychophysics Toolbox (Kleiner et al., 2007), powered by a Macbook Pro, and presented on a 22 inch HP 22vx monitor. During the task, eight identical white disks (0.9 cm diameter equivalent to 1.3° visual angle) were presented against a black background, with targets highlighted by a temporary red outline. Trials varied in the number of targets (2, 3, or 4) and speed of stimulus movement (approx. 7.4, 9.9 or 12.4°s^–1^), the order of which was fully randomized. Performance was assessed using the number of items successfully identified on each tracking trial (as a percentage), based on two blocks of nine trials. Each pre and post-training assessment lasted 5–10 min.

#### Mid-Level Transfer Task

Mid transfer to working memory performance was assessed using the *n*-back task (as used by Jaeggi et al., 2008). The *n*-back task requires participants to decide whether a stimulus in a sequence matches one appearing *n* trials previously. This requires simultaneous storage and manipulation of information, and is proposed to measure working memory capacity (Kane and Engle, 2002). In task 1, spatial *n*-back, a square moving within a 3 × 3 matrix had to be monitored for matches in position with the stimulus appearing three turns previously (i.e., 3-back matches). In task 2, dual spatial and auditory *n*-back, the square had to be monitored for matches in position with the stimulus two turns previous, while *also* monitoring an independent auditory stimulus (spoken letters) for matches two turns previously. Performance was assessed through the percentage of sequence matches correctly identified (a missed match or a false hit were both scored as errors). An overall score was calculated from performance averaged across the two tasks. Each pre and post-training assessment lasted 5–10 min.

#### Far Transfer Task

**FIGURE 2:**
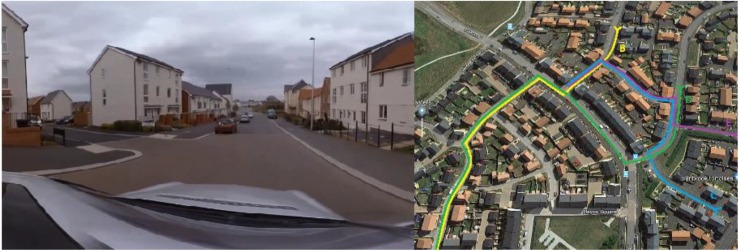
Screenshot from route recall video **(left panel)** and map identification task **(right panel)**.

To assess transfer to a task with multiple demands on working memory, that was considered to be representative of real-world military activities (i.e., with good construct and face validity), participants completed a concurrent route recall and auditory monitoring task. This task was designed to be representative of a vehicle pursuit, where operators would have to attend to multiple sources of information, such as recalling the route taken and monitoring communication devices. Consequently, participants were played a video of a vehicle navigating a housing estate (not exceeding the 30 mph limit) on the large projector screen used in the training task (Figure 2, left panel). They were required to recall the order and direction of all turns made, monitor a stream of sounds for a target sound and count how frequently it occurred, and subsequently identify the route taken on a map showing five possible options (Figure 2, right panel). Dual-tasks such as auditory monitoring have been shown to target working memory (Nissen and Bullemer, 1987; Klingberg, 2000), and holding the specifics of the route requires visual short-term memory. Performance on multitasking tests has also been linked with military performance (Weightman et al., 2015), supporting the link between this type of task and real-world abilities. Performance on this dual visual-auditory task was assessed using an overall score from the three aspects of the recall task (correct counting of auditory tones, correct recall of turns and correct identification of the route on the map) to capture the multitasking requirement of the task, rather than assessing components individually.

### Procedure

The study followed a pre- and post-test design, with random group allocation. On their first visit to the lab all participants completed vision screening tests (Snellen; Ludvigh, 1941) and stereovision tests (Stereo Optical Inc., Chicago, IL, United States) followed by the baseline working memory, near and far transfer tasks and a baseline NeuroTracker assessment. Those allocated to training groups then completed their first training session. The full training group completed five sessions of approximately 30 min, each consisting of four training blocks (i.e., five visits) spread over 12–13 days at 2–3 day intervals. The abbreviated training group completed three training sessions, and the portable group completed five sessions on the tablet device. All groups repeated the baseline tests following training, 12–13 days after baseline assessment.

### Data Analysis

Data was analyzed using 2 (test: pre v post) × 4 (group: full v portable v abbreviated v control) mixed ANOVA. Data was checked for homogeneity of variance (Levene’s test), and skewness and kurtosis. Performance data from the MOT task deviated somewhat from normality (slight negative skew) and was transformed for analyses using a reflected square root transform. Violations of sphericity were corrected for using a Greehouse–Geisser correction factor. No outlying values (more than 3 SD from the mean) were identified.

## Results

### NeuroTracker Performance

**FIGURE 3:**
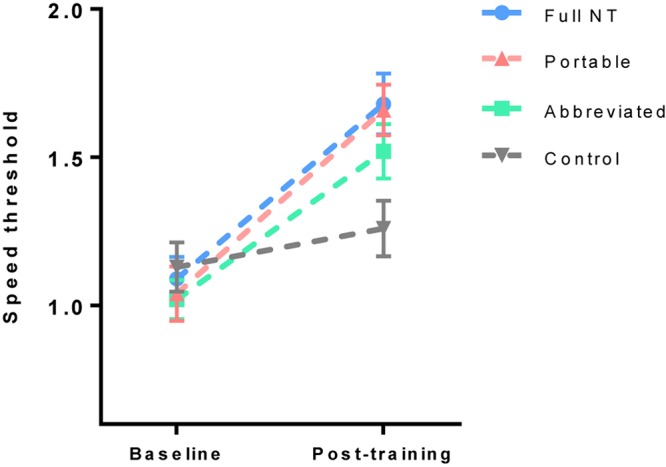
Mean (and standard error) of NeuroTracker speed threshold scores pre and post training.

To assess learning on the training task, a 2 (test) × 4 (group) ANOVA was run on NeuroTracker speed threshold scores. There was a significant main effect of test, *F*(1,80) = 188.61, *p* < 0.001, *η*_p_^2^ = 0.70, but no effect of group, *F*(3,80) = 1.16, *p* = 0.33, *η*_p_^2^ = 0.04. There was also a significant interactive effect, *F*(3,80) = 11.59, *p* < 0.001, η_p_^2^ = 0.30. Bonferroni–Holm corrected *t*-tests showed significant improvements in the full NT group (*p* = 0.004, *d* = 1.65), portable group (*p* = 0.004, *d* = 1.91), and abbreviated group (*p* = 0.004, *d* = 1.97), but not the control group (*p* = 0.051, *d* = 0.45) (Figure 3).

### Near Transfer Task Performance

**FIGURE 4:**
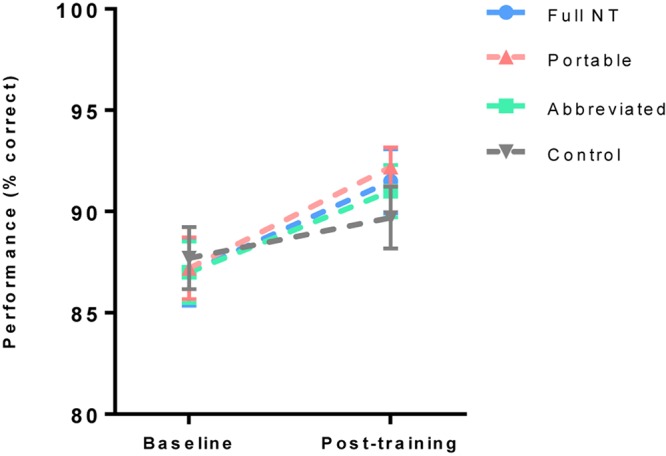
Mean (and standard error) of multiple object tracking (MOT) task performance pre and post training.

To assess improvement on the MOT near transfer task a 2 (test) × 4 (group) ANOVA was run on percentage correct scores from the MOT task. There was a significant increase in performance from baseline to post training, *F*(1,76) = 43.59, *p* < 0.001, *η*_p_^2^ = 0.37, but no effect of group, *F*(3,76) = 1.22, *p* = 0.31, *η*_p_^2^ = 0.05, and no interaction, *F*(3,76) = 0.11, *p* = 0.95, *η*_p_^2^ = 0.00 (Figure 4).

### Mid-Level Transfer Task Performance

**FIGURE 5:**
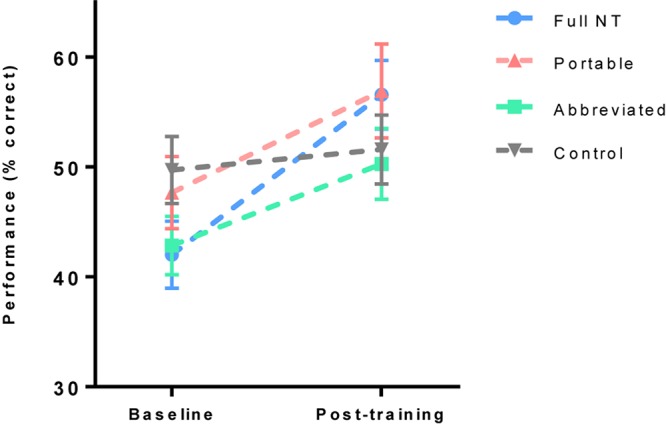
Mean (and standard error) of working memory scores pre and post training.

To assess improvement in working memory performance, a 2 (test) × 4 (group) ANOVA was run on performance scores (% correct) from the combined *n*-back tests (3-back and dual 2-back). Performance was found to be significantly better at post-test, *F*(1,80) = 35.68, *p* < 0.001, *η*_p_^2^ = 0.31. There was no main effect of group, *F*(3,80) = 0.83, *p* = 0.60, *η*_p_^2^ = 0.02, but there was a significant interaction effect, *F*(3,80) = 3.56, *p* = 0.02, *η*_p_^2^ = 0.12. Bonferroni–Holm adjusted *t*-tests showed a significant improvement in WM performance in full (*p* = 0.003, *d* = 1.11), portable (*p* = 0.01, *d* = 0.72) and abbreviated (*p* = 0.02, *d* = 0.62), but not in the control group (*p* = 0.51, *d* = 0.15) (Figure 5).

### Far Transfer Task Performance

**FIGURE 6:**
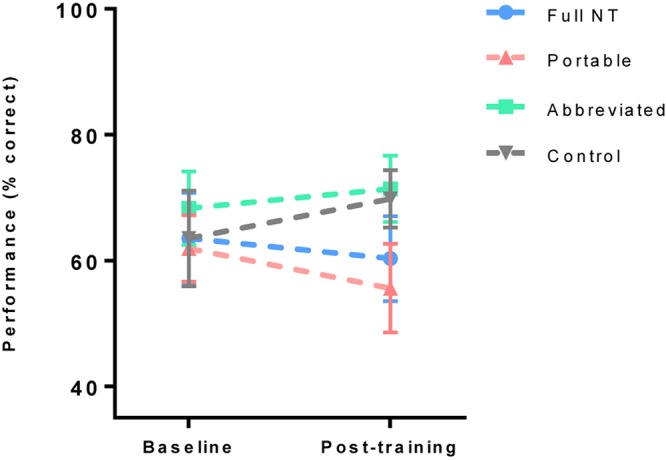
Mean (and standard error) of overall performance on route recall task.

To assess the effect of training group on performance in the far transfer task, a 2 (test) × 4 (group) ANOVA was run on combined route recall scores. There was no effect of test, *F*(1,30) = 0.00, *p* = 1.00, *η*_p_^2^ = 0.00, no effect of training group, *F*(3,80) = 1.00, *p* = 0.40, *η*_p_^2^ = 0.04, and no interaction, *F*(3,80) = 0.55, *p* = 0.65, *η*_p_^2^ = 0.02 (Figure 6), indicating no benefit of NeuroTracker training for the far transfer task.

## Discussion

There is considerable interest in CT from applied researchers and practitioners, as the possibility of developing domain-general abilities to improve cognitive performance is highly attractive in both sporting and military settings (Blacker et al., 2018; Walton et al., 2018; Redick, 2019). This study aimed to address some of the common issues in the CT literature when examining the effectiveness of a CT training intervention (Simons et al., 2016). Near, mid and far transfer measures were assessed pre and post NeuroTracker training to assess how adaptive 3D object tracking training affected object tracking ability, working memory and performance of a real-world dual visual-auditory task.

As expected, NeuroTracker training resulted in significant improvements in NeuroTracker performance across all three training groups. While the control group also trended toward a significant improvement, the effect sizes in the three training groups were considerably larger, indicating a strong training effect. In contrast to our hypothesis, however, there was no effect of training group on performance in the near transfer (MOT) task; a general improvement was seen across all groups which indicates a learning effect from experience with the task. If any transfer effect from NeuroTracker training does exist in this case, it is much smaller than the improvement from repeating the MOT test. Alternatively, the learning effect observed on the NeuroTracker task may have been a result of task specific improvements that were distinct from actual improvements in object tracking ability, hence the lack of transfer to the new MOT task.

As predicted, all three training groups, but not controls, showed significant improvements in working memory performance. There may also have been an effect of training delivery method, as the greatest gains were seen in the full, then portable, then abbreviated groups. In practical terms, this result supports the “dose-response effect” of CT reported by Jaeggi et al. (2008). This finding should, however, be interpreted with some caution, as groups were not well matched at baseline, which may have exaggerated some pre to post differences. For example, the abbreviated training group made a medium to large improvement in performance (*d* = 0.62), but were still no better than controls at post-test, suggesting there could be a regression to the mean effect. Nonetheless, the improvement in WM is in line with previous work, which has shown similar effects of NeuroTracker training (Parsons et al., 2016; Vartanian et al., 2016). Literature on MOT has also strongly indicated WM to be a crucial resource for tracking targets amongst distractors (Allen et al., 2006), with individual differences in WM a significant predictor of tracking performance (Oksama and Hyönä, 2004)^2^. Given the central role played by WM in decision making and performance under pressure (Beilock and Carr, 2005; Furley and Memmert, 2010), WM improvements may represent an important training effect that is worthy of further investigation in the context of military performance (see recommendations of Blacker et al., 2018).

Finally, it was predicted that NeuroTracker training would lead to improved performance on the far transfer task, but there was no evidence of any training benefit, with no general improvement and no group differences. Previous findings have suggested transfer to biological motion perception (Legault and Faubert, 2012) and soccer passing decision making (Romeas et al., 2016) following NeuroTracker training. But overall, the lack of far transfer effect here is in line with the majority of the CT literature (Owen et al., 2010; Stojanoski et al., 2018), despite some positive findings (Biggs et al., 2015; Ducrocq et al., 2018). The more promising findings appear to have been a result of training that is more targeted toward a specific cognitive function relevant to the transfer task. For example, Biggs et al. (2015) and Ducrocq et al. (2016) found benefits of inhibition training for withholding firing on civilians in a shooting simulation, and tennis volley performance under pressure, respectively. In discussing the use of CT for military applications, Blacker et al. (2018) suggest that successful CT may depend on aiming for more “mid-level” transfer effects, where the training task is more closely aligned to the target transfer task, based on effective task analysis. As some researchers suggest that far transfer effects may be unlikely in any form of learning (Sala and Gobet, 2017), this more targeted approach may hold the greatest potential for applied use of CT. Therefore future investigations of adaptive object tracking training may wish to examine transfer to more perceptual, motion tracking tasks that are more similar to the training task, as opposed to the dual visual-auditory task used in this study.

An interesting alternative is proposed in a recent review and meta-analysis by Gathercole et al. (2019). Gathercole et al. propose that transfer from one CT task to another might occur through learning new cognitive routines that must be learnt to complete a new task, rather than by developing the core capacities like WM. They suggest that this could account for the fact that some WM training does not transfer to other WM tasks, yet could still transfer to other tasks that benefit from the newly developed cognitive routine. A mechanism of transfer such as this could explain the benefits for the improved performance on the WM task found here, despite lack of transfer to the other MOT task. If future work supports this proposal, it may offer possibilities for improved use of CT, provided a training task can be used that provides new cognitive challenges relevant to the transfer task.

When interpreting the findings of this study it should be noted that the bespoke transfer task provides both benefits and limitations. The route recall task was developed in consultation with military subject matter experts and was considered to have good face validity, as well as allowing an objective measure of performance. However, as the task was novel, it has not previously been validated and therefore may not provide a fair test of NeuroTracker. Additionally, it may be that group differences after WM training are only revealed under pressure when demands on WM are high (e.g., Ducrocq et al., 2016, 2018). Future research needs to further evaluate the potential of CT to improve performance in challenging environments by developing and validating relevant transfer tasks and using more targeted methods of CT. Similarly, the use of the abbreviated and portable training groups as active controls in this study provided both strengths and weaknesses. As the portable and abbreviated groups provided similar training elements to the full training group, we were able to observe a small “dose-response” effect which suggested that volume of practice and method of delivery were important factors. This indicates that training effects were not simply a Hawthorne effect and are likely to be a specific result of the “active ingredient” in the training. However, as the abbreviated and portable groups faced many similar cognitive demands as the full NT training group, the effect of NeuroTracker training in comparison to any other form of cognitive activity could not be determined. Therefore, future work may benefit from the inclusion of active control tasks that do not include similar cognitive demands to the principle training method.

Future work could also consider the potential of a slightly different tracking task as the basis for CT, one which may have more relevance for tracking real-world objects. While the targets to be tracked in the NeuroTracker task were identical to each other, in a task known as *multiple identity tracking* distinct objects have to be monitored. It appears that fewer targets can be tracked during multiple identity tracking (possibly resulting from serial instead of parallel tracking) and leads to much more frequent gaze fixations directed to targets in a serial fashion (Hyönä et al., 2019; Li et al., 2019). As targets with distinct identities is more akin to, for instance, tracking enemy soldiers or opposition defenders in sport, training based on more realistic targets with distinct identities may have more real-world relevance.

In conclusion, while training on the 3D tracking task led to improvements in working memory, this crucially did not extend to the far transfer test. This finding, which utilized a large sample and addressed several of the shortcomings of CT research, mirrors previous work which suggests CT may benefit other lab based cognitive tests but has limited utility for real-world performance in healthy populations. Future investigations of CT may wish to develop more targeted approaches, aiming for more modest “mid-level” transfer effects (Blacker et al., 2018).

## Data Availability Statement

The datasets generated for this study are available on request to the corresponding author.

## Ethics Statement

The studies involving human participants were reviewed and approved by the School of Sport and Health Sciences Ethics Committee. The patients/participants provided their written informed consent to participate in this study.

## Author Contributions

DH, MW, SS, and SV contributed to the study design. DH and NM conducted the data collection. All authors contributed to the preparation of the manuscript.

## Conflict of Interest

The authors declare that the research was conducted in the absence of any commercial or financial relationships that could be construed as a potential conflict of interest.

## Footnotes

1 www.neurotracker.net

2 An exploratory, *post hoc* analysis comparing participants with high versus low baseline WM (median split), indicated that those high in WM showed better overall NeuroTracker performance (*p* = 0.005, *d* = 0.62) and trended toward better overall MOT performance (*p* = 0.07, *d* = 0.41).
